# Real-Time Radar Classification Based on Software-Defined Radio Platforms: Enhancing Processing Speed and Accuracy with Graphics Processing Unit Acceleration

**DOI:** 10.3390/s24237776

**Published:** 2024-12-04

**Authors:** Seckin Oncu, Mehmet Karakaya, Yaser Dalveren, Ali Kara, Mohammad Derawi

**Affiliations:** 1 TUBITAK BILGEM, Ankara 06100, Turkey; seckin.oncu@tubitak.gov.tr; 2 Department of Electrical and Electronics Engineering, Gazi University, Ankara 06570, Turkey; mehmetkarakaya@gazi.edu.tr (M.K.); akara@gazi.edu.tr (A.K.); 3 Department of Electrical and Electronics Engineering, Izmir Bakircay University, Izmir 35665, Turkey; yaser.dalveren@bakircay.edu.tr; 4 Department of Electronic Systems, Norwegian University of Science and Technology, 2815 Gjovik, Norway

**Keywords:** software-defined radio, electronic support measures, radar classification, parameter extraction, clustering, GPU

## Abstract

This paper presents a comprehensive evaluation of real-time radar classification using software-defined radio (SDR) platforms. The transition from analog to digital technologies, facilitated by SDR, has revolutionized radio systems, offering unprecedented flexibility and reconfigurability through software-based operations. This advancement complements the role of radar signal parameters, encapsulated in the pulse description words (PDWs), which play a pivotal role in electronic support measure (ESM) systems, enabling the detection and classification of threat radars. This study proposes an SDR-based radar classification system that achieves real-time operation with enhanced processing speed. Employing the Density-Based Spatial Clustering of Applications with Noise (DBSCAN) algorithm as a robust classifier, the system harnesses Graphical Processing Unit (GPU) parallelization for efficient radio frequency (RF) parameter extraction. The experimental results highlight the efficiency of this approach, demonstrating a notable improvement in processing speed while operating at a sampling rate of up to 200 MSps and achieving an accuracy of 89.7% for real-time radar classification.

## 1. Introduction

The electronic support measures (ESM) system is a passive surveillance receiver that attempts to identify, analyze, and classify the sources of radar signals. The identification of radar signals plays a critical role in ESM systems. The identification process can be divided into two parts: (a) radar emitter classification (REC) and (b) specific emitter identification (SEI). In REC, radar emitters are categorized based on the statistical analysis of their signal parameters. SEI, on the other hand, focuses on distinguishing identical radar emitters by examining the unique external characteristics of their signals [1,2,3]. Identifying the radar emitters based on pulse description words (PDWs), consisting of radio frequency (RF), pulse amplitude (PA), pulse width (PW), angle of arrival (AOA), and time of arrival (TOA), is a common method of a conventional REC. This transforms the problem into a pattern recognition challenge, making it well suited for machine learning (ML) techniques. Consequently, numerous ML-based methods leveraging the PDW structure of radar signals have been proposed in the literature for REC [4,5,6].

The rapid advancements in radio technology from the 1970s to the 1980s marked a significant shift from analog to digital systems. This transition revolutionized various aspects of radio, including system control, source and channel coding, and hardware design. This paved the way for the software-defined radio (SDR) revolution, which expanded the possibilities of radio-based services by eliminating the limitations imposed by hardware-centric designs. SDR-liberated radio systems from fixed-frequency bands, channel bandwidths, and modulation schemes through the implementation of software-based signal processing enabled unprecedented flexibility and adaptability. This breakthrough was achieved through a combination of technological advancements, including the adoption of multi-band antennas, wideband analog-to-digital converters (ADCs) and digital-to-analog converters (DACs), and the execution of IF, baseband, and bitstream processing functions on general-purpose processors [7,8]. The emergence of SDR as a cornerstone of modern RF signal capture solutions is driven by technological advancements and widespread adoption of low-cost digital signal processors (DSPs) [9]. Unlike traditional radio systems, which rely on hardware for signal processing, SDR systems utilize software, allowing for dynamic reconfiguration and seamless channel selection [10,11].

In recent years, researchers have started to use SDRs in the development of various ESM applications based on the PDW structure of the radar signals [12,13,14]. For instance, in [12], a novel approach was introduced for direction-of-arrival (DOA) estimation, dividing the desired angular sector into a small number of channels. This approach demonstrated the ability to achieve radar detection with reduced size, weight, cost, and computational demands, operating at a sampling rate of 2.4 MSps. Similarly, in [13], an SDR-based ESM system was introduced, employing a pulse detection algorithm aimed at reducing undetected pulses caused by overlaps. It was aimed at mitigating the occurrence of undetected pulses resulting from their overlap, supported by experimental results revealing a sampling rate of 2.4 MSps. Furthermore, in [14], an SDR-based radar detector was shown to process radar signals within a bandwidth of 2.4 MHz, confirming its capability for efficient radar signal detection.

While there is a body of research in the development of various ESM applications based on the PDW structure of the radar signals using SDRs, only one study has investigated the use of SDRs in ML-based REC for ESM systems [15]. This study introduced a scale mixture of normal distributions model for radar emitter classification and clustering. Radar signals were received using an SDR with a sampling rate of 4.17 MSps, but the clustering process was performed offline to analyze the radar signals. This offline approach poses a significant limitation for real-time applications. In fact, in the implementation of the SDR-based REC, there are some significant challenges that need to be addressed [16]. One of them is related to the method that is used for acquiring the signal in real time without losing any samples. In dense and noisy environments, real-time data processing is critical for extracting meaningful information from large volumes of data. Traditional CPU-based solutions, which rely on sequential processing, often struggle with the high computational demands of these scenarios. This can lead to latency, memory bottlenecks, and delays in processing, particularly in time-sensitive applications such as radar systems, telecommunications, and medical imaging. By contrast, GPU-accelerated solutions offer a significant advantage by leveraging parallel processing capabilities. By offloading computationally intensive tasks from the CPU to the GPU, they enable faster, more efficient data handling, drastically reducing latency and improving real-time responsiveness. Additionally, GPU-accelerated FFT optimizes memory management and minimizes data transfers, ensuring quicker processing speeds and higher system performance. These benefits make GPU acceleration particularly valuable for applications where rapid analysis of dense, noisy data streams is essential. Radar classification systems and other data-intensive applications face significant challenges in dense and noisy environments, where real-time processing of vast amounts of high-dimensional data is critical for extracting meaningful information. Traditional CPU-based methods, relying on sequential processing, often struggle with the high computational demands of such scenarios, leading to latency, memory bottlenecks, and delays—issues particularly problematic in time-sensitive applications like autonomous vehicles, defense systems, telecommunications, and medical imaging. GPU-accelerated solutions, with their parallel processing capabilities, offer a promising pathway to overcome these challenges. By offloading computationally intensive tasks from the CPU to the GPU, they enable faster and more efficient data handling, drastically reducing latency, optimizing memory management, and improving real-time responsiveness. These advancements, including GPU-accelerated FFTs for enhanced data transfer efficiency, make GPU acceleration invaluable for rapid analysis in complex and cluttered environments, ensuring timely and accurate performance. To address this challenge, higher sampling rates could be utilized; however, this approach may negatively impact processing speed. Therefore, a cost-effective solution is essential to enhance processing efficiency. Additionally, the complexity of classification algorithms presents another challenge. Employing a robust clustering algorithm is crucial for achieving accurate classification.

In this study, a novel SDR-based radar classification system capable of real-time operation at significantly higher sampling rates compared to existing systems is proposed. To achieve this, a fully functional SDR-based radar classification system implemented in C++ that utilizes the Density-Based Spatial Clustering of Applications with Noise (DBSCAN) algorithm as a classifier was developed. Additionally, Graphical Processing Unit (GPU) parallelization for RF parameter extraction was employed to accelerate the processing pipeline. In the proposed system, the radar signal is first received through the receiver ($R_{x}$) port of the SDR. Then, the envelope of the incoming signal is calculated, and radar parameters are extracted using a thresholding operation. Next, the samples within a pulse are transferred to the GPU for parallel RF parameter extraction. Finally, DBSCAN algorithm that takes PDWs as input is employed to perform radar classification.

The primary contributions of our work are twofold and are listed as follows:

- A real-time radar classification system capable of operating at a sampling rate of up to 200 MSps was implemented using an efficient signal processing algorithm to exceed the capabilities of existing systems.
- GPU parallelization for RF parameter extraction was employed to reduce the computational burden that allows for real-time operation with a high sampling rate.

The rest of the article is structured as follows. The materials and algorithms used to develop the proposed system are presented in Section 2. Then, the experimental setup is described in Section 3. Next, the classification results are presented in Section 4. Lastly, Section 5 concludes the paper with a summary of the contributions and future directions.

## 2. Materials and Methods

### 2.1. Radar Classification in ESM

**Figure 1:**
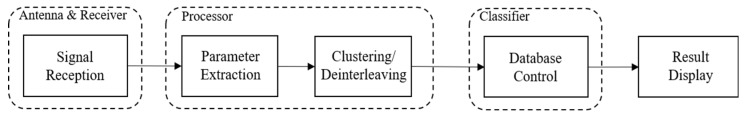
Illustration of a functional diagram of an ESM system.

The primary function of an ESM system is to detect threats and conduct surveillance of a specific area to identify the sources of electromagnetic emissions. This system is designed to analyze the pulse-by-pulse measurements obtained by the receiver, enabling it to identify known radars and indicate their presence [17,18,19,20]. As depicted in Figure 1, the ESM system comprises several key components. The antenna captures RF signals from the surrounding environment and directs them to the receiver. The receiver digitizes the incoming RF signals, converting them into a format suitable for further processing. The processor measures the pulse parameters and extracts relevant features, transforming the pulses into PDWs. PDWs encapsulate interpulse parameters such as TOA, RF, PW, and PA. The deinterleaving stage separates overlapping pulses, while the clustering stage groups pulses based on their similarities. The classifier compares the extracted PDWs against the emitter database, identifying the corresponding radar type or classifying the signal as unknown if no match is found. The classification results are presented to the user in a tabular or graphical format for easy interpretation and understanding [21,22,23].

In the following sections, the details of SDR as a receiver system, including the parameter extraction process, and the clustering/deinterleaving process are discussed.

### 2.2. SDR Architecture

Conventional radios employ dedicated hardware components to perform specific functions, such as signal modulation/demodulation, data encoding/decoding, and more. These specialized hardware components handle all aspects of signal processing. However, SDR technology introduces a paradigm shift by re-placing certain traditional radio components with software-based implementations [24].

SDR architecture offers several significant advantages. These include flexibility, as SDR systems empower the alteration of receiver functionality via software, facilitating dynamic reconfiguration and adaptation to diverse signal types and communication protocols. Furthermore, SDR systems have the potential to reduce hardware costs by replacing specialized hardware components with software-based implementations. In addition, SDR systems are scalable, offering the ability to adjust to changing bandwidth and processing requirements. Finally, SDR systems are built on open standards, promoting interoperability and compatibility with a wide range of software and hardware components. By exploiting the advantages of software-defined radio (SDR) architecture, SDR technology has significantly transformed the field of radio communication, offering a highly versatile and adaptable platform for signal processing and receiver design. The inherent flexibility and reconfigurability of SDR systems have enabled a wide range of applications, such as wireless communication and radar systems [25,26]. However, SDR systems remain vulnerable to performance degradation due to electromagnetic interference (EMI). In particular, the sensitivity of the SDR receivers, a key parameter determining their ability to detect weak signals, can be adversely affected by EMI.

The intricate interaction between EMI sources and receiver components can result in unpredictable fluctuations in sensitivity. Although shielding materials can help mitigate EMI, effectively capturing transient and non-periodic EMI signals remains a significant challenge. To ensure optimal performance, it is necessary to employ proper strategies to reduce the impact of EMI on HF SDR receiver sensitivity, focusing on design considerations, shielding techniques, and advanced signal processing algorithms [27].

The SDR receiver consists of two primary components: the analog front-end and the DSP components. The analog front-end handles the narrowband frequency down-conversion and subsequent ADC. The down-conversion process shifts the incoming signal from a high frequency band to a lower IF band, while the ADC converts the analog IF signal into a digital representation suitable for further processing. The DSP components handle the remaining signal processing tasks, including demodulation, filtering, and channel decoding. These operations are performed in the digital domain. The DSP components utilize software algorithms to manipulate the digital signal representation, enabling flexible and reconfigurable signal processing capabilities.

**Figure 2:**
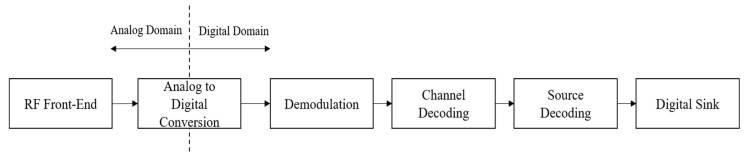
Functional structure of an SDR receiver.

The data flow in an SDR receiver system is depicted in Figure 2. As shown in the figure, first, the RF front-end initiates the signal processing chain by down-converting the intercepted RF signal to a baseband frequency. This down-conversion process translates the signal from its original high-frequency band to a lower IF band, enabling it to be sampled and processed by an ADC. After conversion to the digital domain, the demodulation process extracts the underlying information from the modulated electromagnetic waveform, transforming it into its corresponding binary representation. Subsequently, a channel decoder removes the controlled redundancy introduced during data transmission, restoring the binary information to its original state [24,28].

In the RF front-end stage, SDR commonly utilizes direct conversion receiver (DCR) architecture. Due to their inherent advantages over superheterodyne receivers, DCRs have garnered widespread popularity. The key characteristic of DCR architecture is its compact design, achieved by employing a single mixing stage that directly translates the frequency from RF to baseband. This direct down-conversion approach allows for signal amplification and filtering at baseband, leading to reduced power consumption and simplified image rejection. These advantages collectively contribute to the implementation of the entire receiver as a monolithic integrated circuit, reducing manufacturing costs [29].

**Figure 3:**
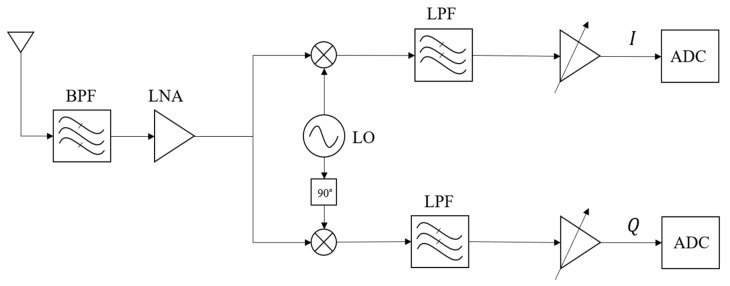
IQ Implementation of DCR.

A typical DCR architecture is illustrated in Figure 3. The incoming RF signal is first filtered and amplified to ensure optimal signal quality. Then, the signal undergoes direct down-conversion with a local oscillator (LO) signal, generating an IF signal centered at baseband. Next, the IF signal is further filtered to remove unwanted frequency components and prepare it for ADC conversion. The ADC converts the analog IF signal into a digital representation, enabling further processing in the digital domain [30].

Following the initial filtering and amplification stages, the RF signal undergoes down-conversion to baseband using a pair of mixers and quadrature sinusoids, which combine to form a complex sinusoid. This complex sinusoid, representing the down-converted signal, is then subjected to filtration through two low-pass filters, one in the I-channel (in-phase) and the other in the Q-channel (quadrature). The down-conversion process utilizes the concept of heterodyning, where the incoming RF signal is mixed with a LO signal. The resulting mixed signal contains both sum and difference frequencies. By choosing an appropriate LO frequency, the desired signal is translated to baseband, while unwanted frequency components are filtered out. The quadrature sinusoids, represented as sine and cosine waveforms, are employed to extract the I and Q components of the down-converted signal. These components correspond to the real and imaginary parts of the complex sinusoid, respectively. The I and Q components are then passed through individual low-pass filters to suppress out-of-band noise and further refine the baseband signal. The combination of mixers, quadrature sinusoids, and low-pass filters enables the DCR architecture to efficiently down-convert the RF signal to baseband while maintaining signal integrity and reducing the impact of noise. This process forms the foundation for subsequent signal processing stages in the SDR receiver [31].

A real bandpass signal, *x*(*t*), can be mathematically represented as follows [32]:

(1) $$x\left(t\right)=r\left(t\right)cos(2\pi f_{0}t+\Phi_{x}\left(t\right)),$$

where $r\left(t\right)$ is the signal’s envelope, $\Phi_{x}\left(t\right)$ is its time-varying phase, and $f_{0}$ is the carrier frequency. It is important to note that both $r\left(t\right)$ and $\Phi_{x}\left(t\right)$ have frequency components that are significantly lower than $f_{0}$.

Bandpass signals can be equivalently represented by two lowpass signals, known as quadrature components, using the complex envelope representation as shown below:

(2) $$x\left(t\right)=x_{I}\left(t\right)cos2\pi f_{0}t-x_{Q}\left(t\right)sin2\pi f_{0}t.$$

Here, $x_{I}\left(t\right)$ and $x_{Q}\left(t\right)$ are real lowpass signals known as the in-phase and quadrature components, respectively, and are defined by the following:

(3) $$x_{I}\left(t\right)=r\left(t\right)cos\Phi_{x}\left(t\right),$$

(4) $$x_{Q}\left(t\right)=r\left(t\right)sin\Phi_{x}\left(t\right).$$

Following this, the I  and Q branches are sampled using two ADCs. The paired samples are then combined and stored as complex numbers.

### 2.3. Pulse Parameter Extraction

**Figure 4:**
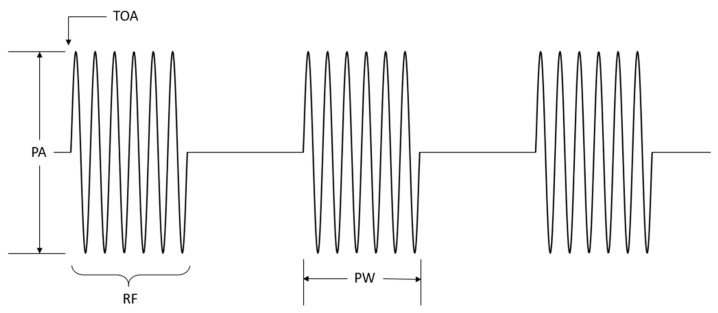
Illustration of radar pulse and some basic parameters.

The extraction of radar pulse parameters plays a crucial role in the signal processing flow in ESM receivers. Accurately extracting individual pulse intercepts from the interleaved pulses emitted by multiple radars along with identifying the corresponding radar sources hinges on the precise measurement of fundamental pulse parameters. The ESM receiver measures the PDWs, consisting of RF, PW, PA, and TOA for each pulse [33]. The parameters of radar pulse are illustrated in Figure 4.

In the proposed system, SDR is employed as the ESM receiver to capture the radar signals. As described in Section 2.2, digitized IQ data serves as the input for pulse parameter extraction. To extract these parameters, the envelope of the signal is calculated to determine the amplitude of each sample. From Equations (1), (3) and (4), the envelope of IQ signal, ($r\left(t\right)$), is represented as follows:

(5) $$r\left(t\right)=\sqrt{x_{I}{\left(t\right)}^{2}+x_{Q}{\left(t\right)}^{2}}.$$

The TOA is determined as the sample count when the current sample amplitude exceeds the predefined threshold while the preceding sample amplitude falls below the threshold. Dividing this value by the sampling frequency ($f_{s}$) yields the TOA in seconds. Conversely, the time of departure (TOD) is identified as the sample count when the current sample amplitude falls below the threshold while the preceding sample amplitude surpasses the threshold. Dividing this value by $f_{s}$ yields the TOD in seconds. This method effectively captures the TOA and TOD of individual pulses, enabling the extraction of pulse parameters for subsequent analysis. PW is obtained by difference in TOD and TOA.

The pulse amplitude (PA), on the other hand, can be obtained by

(6) $$PA=\sum_{i=0}^{N}\frac{r_{i}\left(t\right)}{N},$$

where N represents the total number of samples within the pulse, and $r_{i}\left(t\right)$ refers to each individual sample in the pulse.

Frequency, another crucial parameter to extract from pulse information, can be determined using various methods. In this study, two techniques were employed: Fast Fourier Transform (FFT) and Instantaneous Frequency Measurement (IFM). The FFT is a computational algorithm that efficiently calculates the Discrete Fourier Transform (DFT) of a signal. The DFT, a mathematical transformation, reveals the frequency components embedded within a signal or a set of data.

The DFT ($X\left(k\right)$) of a finite-length sequence x(n) is represented by a set of N frequency domain coefficients as follows [32]:

(7) $$X\left(k\right)=\sum_{n=0}^{N-1}x\left(n\right)e^{-\frac{j2\pi nk}{N}};\;k=0,\ldots ,N-1.$$

When employing an N-point FFT, the input frequency band is effectively divided into N bins, each with a width of $f_{s}/N$. Consequently, the FFT operation can be applied to each incoming sample in the pulse, resulting in a spectrum representation of the signal. After FFT implementation, the FFT bin with the highest magnitude corresponds to the dominant frequency component of the signal.

The frequency bin with the peak magnitude, denoted as $k_{max}$, corresponds to the index where the magnitude of the DFT is maximal. It can be expressed as follows:

(8) $$k_{max}={argmax}_{k}\left|X(k)\right|.$$

where  X(k) is the DFT of the sequence x(n), $\left|X(k)\right|$ is the magnitude of the frequency bin at index k, and ${argmax}_{k}$ denotes index k where $\left|X(k)\right|$ is maximized. After identifying $k_{max}$, the subsequent step involves calculating the frequency shift ($f_{shift}$) of that bin relative to the sampling frequency. The center frequency for any frequency bin *k* in the DFT can be calculated using $f_{k}=\frac{k}{N}f_{s}$. Here, *f_k_* represents the frequency associated with bin *k*, *N* is the total number of samples in the DFT, and *f_s_* is the sampling frequency. The frequency shift is then the difference between this frequency and the center of the frequency spectrum and is given by

(9) $$f_{shift}=\frac{k_{max}*f_{s}}{N}.$$

Then, the pulse frequency can be found as

(10) $$f_{pulse}=f_{0}+f_{shift}.$$

The terms $f_{0}$, $f_{shift}$, and $f_{pulse}$ represent the original or center frequency of the pulse signal (often the carrier frequency in modulation), the change in frequency due to a time shift or modulation, and the observed pulse frequency after the shift, respectively. The IFM is a signal processing technique that estimates the instantaneous frequencies of multiple concurrent signals. This method involves analyzing the autocorrelation of the received signals to determine their instantaneous frequency variations over time. Once the I and Q samples are obtained, the phase information can be extracted by applying the following relationship:

(11) $$\Phi_{x}\left(t\right)=\tan^{-1}\frac{x_{Q}\left(t\right)}{x_{I}\left(t\right)}.$$

In the next, the instantaneous frequency, or modulation frequency, $f_{m}\left(t\right)$, can be calculated from

(12) $$f_{m}\left(t\right)=\frac{1}{2\pi }\frac{d}{dt}\Phi_{x}(t).$$

In this case, the expression provided in (10) can be restated as

(13) $$f_{pulse}=f_{0}+f_{m}(t).$$

FFT and IFM are two distinct signal processing techniques with unique operational principles and application domains, each offering specific advantages and limitations. The FFT is a mathematical algorithm that transforms a time-domain signal into its frequency-domain representation, enabling a detailed analysis of the spectral content of a signal over a specified time window. This frequency-domain analysis provides insights into the amplitude and phase of each frequency component, making FFT particularly useful for applications that require high-resolution spectral analysis, such as communication systems [34]. However, the accuracy and resolution of FFT are influenced by the length of the time window and sampling rate, with longer windows providing higher-frequency resolution but potentially missing transient signal changes. Moreover, FFT processing is computationally intensive, which can limit its application in real-time systems with strict latency constraints.

By contrast, the IFM technique is designed for the rapid detection of a signal frequency at any given moment, making it well suited for real-time radar applications, where prompt frequency estimation is crucial [35]. IFM provides continuous, near-instantaneous frequency tracking, offering a quick response to frequency variations within a signal. Unlike FFT, which analyzes the entire frequency spectrum, IFM typically focuses on identifying the dominant instantaneous frequency, making it highly efficient in terms of processing speed and suitable for dynamic signal environments. However, the accuracy of IFM can be significantly affected by noise, as it lacks the detailed spectral filtering capability inherent in FFT. This sensitivity to noise may lead to inaccuracies in frequency estimation, particularly in complex or noisy environments.

In summary, the choice between FFT and IFM depends on the specific application requirements, including the need for frequency resolution, processing speed, and noise resilience. FFT is preferable for high-resolution spectral analysis when processing time is not a primary constraint, while IFM is ideal for applications demanding rapid, real-time frequency tracking, albeit with potential trade-offs in accuracy under noisy conditions. Thus, selecting the appropriate technique requires careful consideration of signal characteristics, desired information, and operational constraints [36].

### 2.4. GPU Accelerated FFT

The advent of GPU-accelerated FFT has revolutionized signal processing, particularly for handling large datasets. Traditionally, FFT computations rely heavily on the Central Processing Unit (CPU), causing memory bottlenecks and hindering the execution of other critical tasks. By utilizing the GPU’s parallel processing power, GPU-accelerated FFT offloads the computationally intensive FFT operations from the CPU, freeing up resources for other essential calculations. This approach not only enhances the overall processing speed but also optimizes memory allocation and minimizes data transfers between the CPU and GPU [37,38].

The NVIDIA CUDA Fast Fourier Transform library (cuFFT) provides a streamlined interface for efficient FFT computation on NVIDIA GPUs, eliminating the need for customized GPU FFT implementations [39]. cuFFT streamlines the process of utilizing the GPU’s powerful floating-point capabilities and parallel processing architecture to accelerate FFT computations. This library offers a user-friendly interface that allows developers to quickly harness the GPU’s parallel processing power while benefiting from a highly optimized and thoroughly tested FFT implementation [40,41,42].

The cuFFT algorithm is built upon FFTW (Fastest Fourier Transform in the West), a widely used and highly efficient CPU-based FFT library [43]. cuFFT inherits FFTW’s effectiveness and provides a straightforward plan configuration mechanism that optimizes the FFT operation for the specific GPU hardware and configuration. In this study, 1D DFT was employed, entailing both forward and backward computations. Forward DFT computation stores positive frequencies in the first half of the output, while backward DFT computation stores negative frequencies in the first half of the output. Then, using (7), backward DFT converts a frequency-domain representation back to the original time-domain signal.

### 2.5. Clustering with DBSCAN Algorithm

Radar pulse clustering is a crucial technique for extracting meaningful information from radar signals. Among various clustering algorithms, density-based spatial clustering of applications with noise (DBSCAN) maintains its popularity due to its effectiveness in handling noise, outliers, and varying densities within the data. Unlike traditional clustering algorithms that rely on predefined cluster shapes or centroids, DBSCAN identifies clusters based on the local density of data points, making it robust to noise and variations in cluster shapes. Moreover, the ability of DBSCAN to identify the clusters of arbitrary shapes and sizes makes it well suited for analyzing radar pulse data, which often exhibit non-uniform densities and shapes [44,45,46].

DBSCAN operates by iteratively examining data points and assigning them to clusters based on their neighborhood density. The algorithm requires two input parameters—the maximum distance between two data points ε (eps) and a minimum number of points (Nmin) required to form a dense region—for a cluster. Starting with an arbitrary data point, DBSCAN checks if its ε-neighborhood contains at least Nmin points. If so, the data point and its neighbors are considered part of the same cluster. The process continues until all data points have been visited and assigned to clusters or labeled as noise [39]. Utilizing the principles outlined in [44,45,46,47], the DBSCAN algorithm was implemented in this study.

The ε-neighborhood of a point x is defined based on a dataset D and a distance function $d\left(.,.\right)$ as

(14) $$N_{\varepsilon }\left(x\right)=\left\{y\in D:d\left(x,y\right)\leq \varepsilon \right\}.$$

If the count of neighbors within this neighborhood exceeds a threshold, $N_{min}$, the data point and its neighbors form a new cluster. Following this, additional neighbors are iteratively identified for each unclustered neighbor within the ε-neighborhood.

A point x is considered directly density-reachable from a point y (with respect to ε and $N_{min}$), if x belongs to the neighborhood $N_{\varepsilon }\left(y\right)$ ($x\in N_{\varepsilon }\left(y\right)$) and the neighborhood $N_{\varepsilon }\left(y\right)$ contains at least $N_{min}$ ($\left|N_{\varepsilon }\left(y\right)\right|\geq N_{min}$). Here, $\left|N_{\varepsilon }\left(y\right)\right|$ represents the total number of points within the ε-neighborhood of y.

Data points within the ε-neighborhood of a query point are included in cluster if the number of such points exceeds $N_{min}$, thereby forming a density-connected region. Two points, x and y, are considered density connected with respect to ε and $N_{min}$ if there exists a point z such that both x and y are density reachable from z with respect to ε and $N_{min}$. This indicates that a sequence of density-reachable points links x and y, where each point in the sequence has at least $N_{min}$ neighbors within the ε-neighborhood [47].

Let D be a dataset. A cluster C with respect to ε and $N_{min}$ is defined as a non-empty subset of D that meets the following conditions:

- ∀x,y∈D, if x∈C and y is density reachable from x with respect to ε and $N_{min}$, then y∈C.
- ∀x,y∈C, x and y must be density connected with respect to ε and $N_{min}$.

Among the various parameters available for radar signal clustering, AOA, RF, and PW are considered the most reliable for identifying and distinguishing different radar sources [48,49]. Given the capabilities of our single-channel system, extracting AOA information is not feasible. Therefore, in this work, the DBSCAN algorithm was employed to cluster PDW data using RF and PW parameters as the primary features. This approach effectively utilizes the available information to group radar signals based on their RF and PW characteristics, enabling us to identify and distinguish different radar sources.

## 3. Experimental Evaluation

### 3.1. Hardware Setup

**Figure 5:**
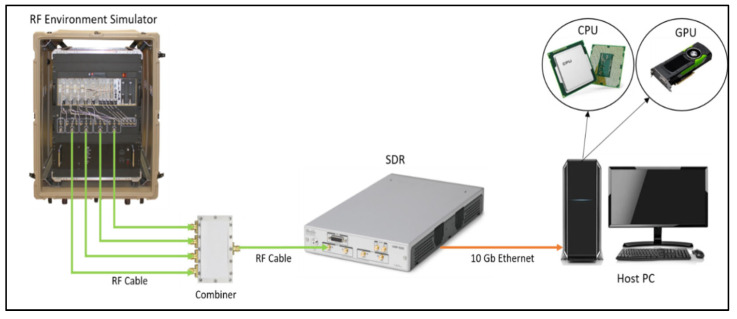
Illustration of the experimental setup.

Figure 5 depicts the experimental setup employed for real-time classification of radar signals utilizing SDR technology. The radar signals are generated using a sophisticated commercial environment simulator, which serves as a crucial test and evaluation tool for the design and analysis of advanced radar receiver systems. This simulator generates realistic scenarios that replicate various multi-threat radar environments within a controlled setting, ensuring precision and reproducibility. An RF combiner is used to merge the RF signals emanating from various ports of the environment generator.

The SDR is positioned to receive the RF signals through its $R_{x}$ port with an RF cable connecting the SDR to the combiner. Subsequently, the SDR undertakes the critical task of digitizing the received RF signal, converting it into IQ data. These IQ data are then transmitted to the host PC via a dual 10-gigabit Ethernet interface for further analysis and real-time classification. This experimental setup offers a reliable platform for systematically evaluating and classifying radar signals, combining the power of SDR technology with an advanced RF Environment Simulator to create realistic and controlled radar scenarios. In this work, Ettus USRP N320 SDR [50] is employed as the SDR platform, which features a frequency range of 3 MHz to 6 GHz with an instantaneous bandwidth of 200 MHz per channel.

As described in previous sections, both the FFT and IFM methods were employed for frequency estimation. The parameters of the SDR receiver and the generated test data are presented in Table 1 and Table 2, respectively. The RF environment generator created the test data, which were then processed by the SDR. Within the SDR framework, a comparative analysis was conducted by performing FFT computations on both CPU and GPU platforms, as well as IFM computation on the CPU. The IFM computation on the GPU was intentionally excluded from this analysis due to its relatively fast processing times.

**Figure 6:**
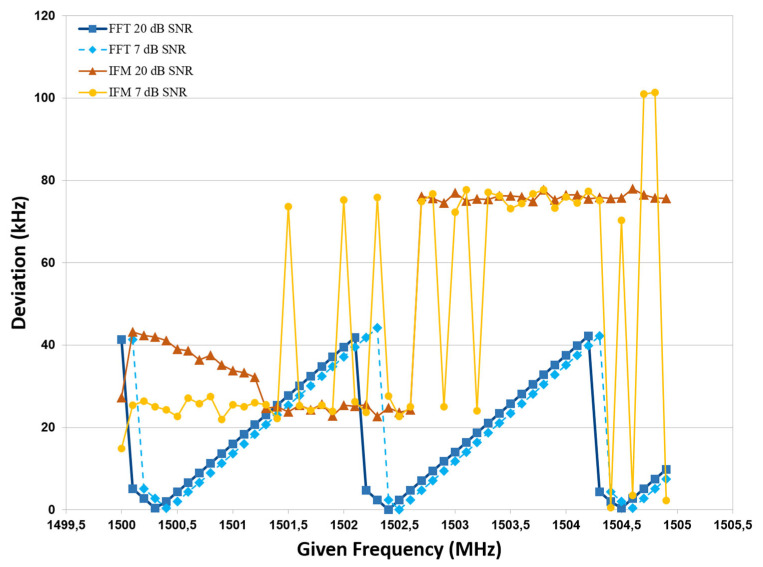
Frequency measurement results of test scenario.

Some of the results regarding the frequency measurement are presented in Figure 6. A comparative analysis of the data reveals that the FFT algorithm delivers results with a higher degree of accuracy than those obtained from the IFM method. The FFT results generally meet expectations, with an estimated accuracy of around 95% to 100%. However, there is insufficient information to quantify the deviations in the IFM values as a percentage. This is particularly evident in the presence of signal noise, where the FFT algorithm exhibits remarkable robustness in yielding results that align with anticipated outcomes. This resilience stems from the inherent correspondence between the FFT bins and the frequency components of the signal, enabling the algorithm to effectively mitigate the impact of noise and provide reliable spectral information. Therefore, the FFT algorithm demonstrates enhanced resilience, particularly in the face of low signal-to-noise ratio (SNR) values.

To evaluate the algorithms’ performance in terms of speed, different $f_{s}$ values were implemented, and a continuous RF signal with the same parameters as outlined in Table 1 was applied. The packet drop rate was monitored as the signal was received by the SDR. As the packet drop rate increases, there is a corresponding increase in processing time. The results are summarized in Table 3.

Based on the experimental results, the FFT algorithm was selected due to its superior robustness. Although it exhibits slower CPU performance compared to the IFM method, we opted to implement the FFT algorithm on a GPU utilizing the cuFFT library. This approach effectively eliminated sample dropping errors even at peak capacity.

### 3.2. Software Design

The core code implementation is structured as an iterative process within an infinite loop. The primary goal is to extract pulsed radar parameters through a multi-step procedure outlined in previous sections. Initially, the signal is subjected to envelope extraction to identify pulse characteristics. Once pulses are detected, the corresponding samples are transferred to the GPU for FFT computation using the cuFFT library. The resulting pulse parameters are recorded in a PDW array. Upon reaching 100 entries in the PDW array, the DBSCAN clustering algorithm is employed to identify distinct clusters within the radar signals. Simultaneously, the PDW array is reset to clear previous data, and associated counters are reset to maintain data integrity.

**Figure 7:**
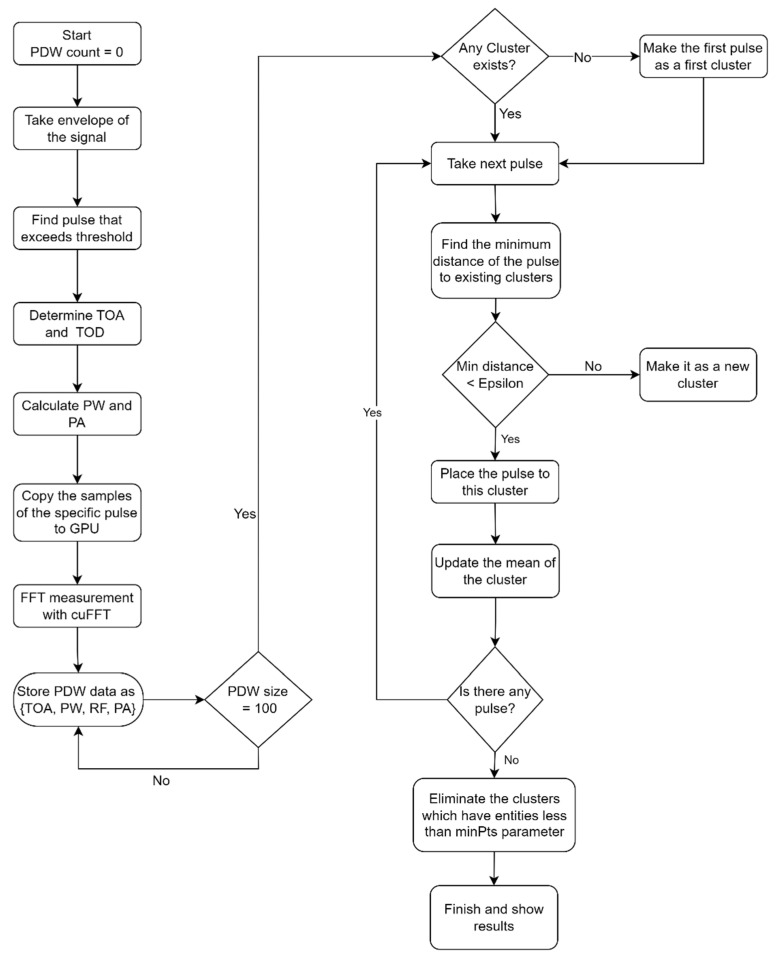
Flowchart of the proposed radar classification algorithm.

A comprehensive flowchart, shown in Figure 7, illustrates the intricate steps of the radar classification algorithm, providing a visual representation of the parameter extraction and clustering procedure. This iterative and parallelized approach enables efficient extraction and analysis of critical radar signal information, facilitating subsequent stages of data interpretation and decision making within the radar processing framework.

Initially, the algorithm sets the PDW count to zero for each new data stream. Following this, the envelope of the incoming signal is extracted, a critical step that emphasizes the waveform shape, facilitating the identification of individual pulses.

In the pulse detection phase, the algorithm identifies pulses that exceed a predefined threshold. For each detected pulse, the TOA and TOD are determined, providing temporal markers that allow for accurate temporal characterization. Subsequently, the PW and PA are calculated, which are essential parameters for pulse characterization and later clustering stages.

For frequency analysis, the samples of the detected pulse are copied to the GPU for rapid processing. An FFT is then applied using cuFFT, enabling a detailed examination of the frequency components of the pulse. The algorithm stores the PDW data in the format TOA, PW, RF, or $f_{pulse}$, PA, until a batch size of 100 PDWs is accumulated, at which point the data batch undergoes further processing. This batch-wise approach optimizes processing efficiency and maintains system organization.

After frequency analysis, the clustering process is followed. If no clusters exist, the algorithm designates the first detected pulse as the initial cluster. For each subsequent pulse, the algorithm calculates the minimum distance to existing clusters. If this minimum distance is below a defined threshold (ε), the pulse is assigned to the nearest cluster. Otherwise, the pulse forms a new cluster. When a pulse is assigned to an existing cluster, the mean of the cluster is updated to incorporate the new data, ensuring that the cluster parameters remain representative of all included pulses.

The algorithm continues processing until no further pulses are available. Once the input stream is exhausted, it eliminates clusters that contain fewer entities than a specified threshold (minPts), effectively filtering out clusters likely representing noise or insignificant data points. Finally, the algorithm concludes by displaying the final clustering results, making them available for interpretation or further analysis.

## 4. Results and Discussions

**Figure 8:**
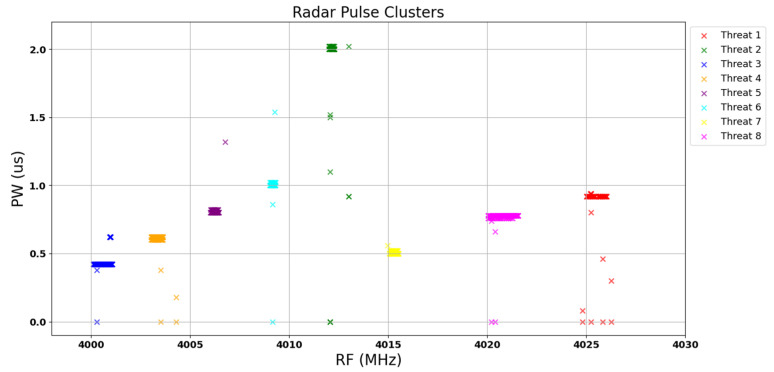
Clusters in PW-RF plane.

**Figure 9:**
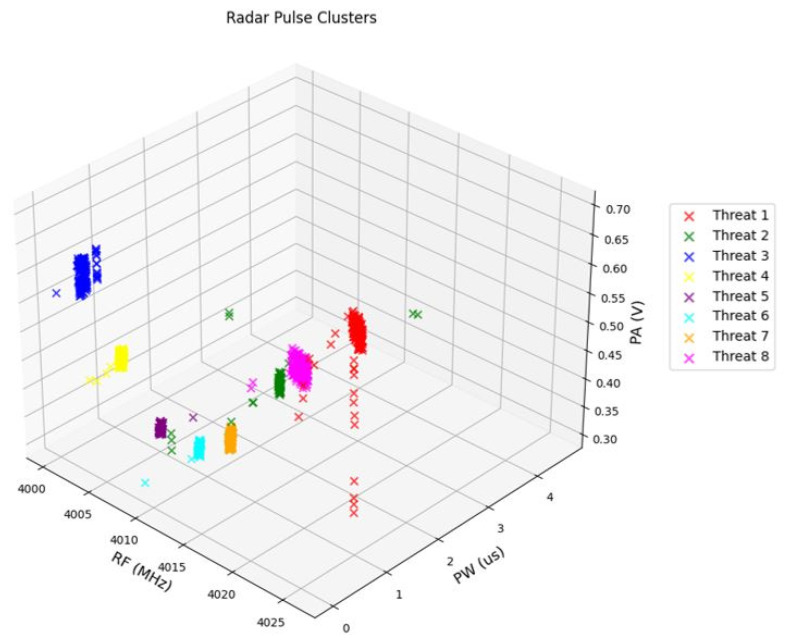
Clusters in PW-PA domain.

In this section, the proposed system is evaluated using the acquired dataset. The testing configurations are detailed in Table 4, while the threat parameters used for this scenario and assumed to exist in the environment are shown in Table 5. Gaussian-distributed errors were introduced to the RF parameters to illustrate the dispersion of samples within the cluster. The operational sequence of each radar was dependent on the emission of a predetermined number of pulses by its predecessor, as outlined in Table 3. The culmination of this sequential radar operation is presented in a two-dimensional representation, specifically a diagram illustrating the relationship between RF and PW parameters, as explained in previous sections. This clustering result, which is visualized in Figure 8, encapsulates the emergent patterns and associations within the radar data, providing a comprehensive portrayal of the interplay between RF and PW parameters across the radar network. Moreover, the parameters PW, RF, and PA are shown in Figure 9. As can be observed, the radar signals were identified as follows: Radar 1 as Threat 3, Radar 2 as Threat 4, Radar 3 as Threat 5, Radar 4 as Threat 6, Radar 5 as Threat 2, Radar 6 as Threat 7, Radar 7 as Threat 8, and Radar 8 as Threat 1.

After the clustering stage, it was determined that 929 pulses were assigned to distinct clusters. The visual representations, provided in Figure 8 and Figure 9, confirm the accurate clustering of all eight distinct radars, highlighting the effectiveness of the clustering methodology. Upon closer examination of the clusters, a detailed analysis revealed that 32 pulses were erroneously assigned to clusters. Nevertheless, the overall accuracy of the clustering process remained remarkable, with a total of 897 pulses out of the initial 1000 pulses demonstrating accurate assignment to their respective clusters. This also indicates the robust performance of the clustering algorithm in successfully categorizing the majority of pulses, thereby validating its efficiency within the context of radar signal processing.

## 5. Conclusions

In this work, an SDR-based real-time radar classification system was developed and implemented. The intricate design and operational details of this system were comprehensively described throughout the paper. Furthermore, a rigorous performance evaluation was conducted, providing a thorough assessment of its effectiveness and capabilities in the realm of real-time radar signal classification. The strategic decision to offload resource-intensive FFT computations to the GPU for parallel frequency parameter estimation was proven to be an effective approach. This segregation optimized resource utilization, alleviating the computational burden on the CPU and consequently enhancing overall processing speed for concurrent operations. As a result, the sampling rate of the radar classification algorithm was successfully increased, thereby significantly improving its processing capabilities.

In the future, our primary focus will be centered on the integration of a TOA-based deinterleaving algorithm, coupled with the implementation of a database query system specifically designed for threat identification within a library context. This multifaceted approach aims at enhancing the effectiveness of threat detection mechanisms in an operational environment. Furthermore, we will broaden our focus to include a thorough examination and management of Pulse-on-Pulse phenomena, particularly in radar-rich environments characterized by high signal density. This strategic consideration is poised to strengthen the robustness of our system in scenarios where overlapping radar pulses pose intricate challenges to conventional signal processing methodologies.

Future work could also extend the current experimental setup by integrating RFNoC for FPGA-based pre-processing. This integration would leverage the parallel execution capabilities of FPGAs, improving the system efficiency in managing high-speed radar signal processing tasks. Offloading the initial signal processing stages to the FPGA would reduce the computational load on the GPU, resulting in better resource utilization and potentially faster processing speeds. Furthermore, FPGA-based pre-processing could enable real-time processing of more complex radar signals with greater scalability. This approach would enhance overall system performance, enabling the classification of a broader range of radar signals with greater accuracy and reduced latency. Further studies could explore the implementation of various FPGA architectures and evaluate the trade-offs between FPGA and GPU-based systems in terms of performance, energy consumption, and flexibility. On the other hand, as the authors have focused on computational aspects, some alternative clustering methods can be considered in future work for improving accuracy, independent from computational aspects.

## Figures and Tables

**Table 1:** SDR receiver configuration.

Sampling Rate	Center Frequency	FFT Size
10 MSps	1502.5 MHz	1024

**Table 2:** Test data.

Number of Pulses	Frequency (MHz)	PW (µs)	Duty Cycle (%)	SNR (dB)
50	1500–1505 MHz	1024	10	7; 20

**Table 3:** Dropped packet performance of IFM and CPU/GPU FFT.

Sampling Rate	IFM	FFT (N= 1024) on CPU	FFT (N=1024) on GPU
50 MSps	0	0	0
100 MSps	0	10	0
200 MSps	20	50	0

**Table 4:** Setup configuration.

Sampling Rate (Msps)	Center Frequency (MHz)	FFT Tap Size	Distance (ε)	Data Points ($N_{min}$)
50	4013	1024	1	5

**Table 5:** Threat parameters.

Radar No.	Number of Pulses	RF ^1^ (MHz)	PW (µs)	PRI (µs)	PA (dBm)	PRI Type
1	19	4000	0.4	113–317–419	−17	Staggered
2	18	4003	0.6	89–199	−20.1	Dwell-Switch (9 pulses)
3	10	4006	0.8	211	−23	Constant
4	10	4009	1	271	−25	Constant (±%3 jittered)
5	10	4012	2	547	−22	Constant
6	20	4015	0.5	73–101–103	−22	Staggered
7	20	4020	0.75	337–457	−19	Dwell-Switch (5 pulses)
8	20	4025	0.9	991	−18	Constant

^1^ RF and $f_{\mathrm{pulse}}\;\mathrm{can}\;\mathrm{be}\;\mathrm{interchangeably}\;\mathrm{used}$.

## Data Availability

The data presented in this study are available on request from the corresponding author.
